# Management of endocrine emergencies: joint consensus statement for management of myxoedema coma

**DOI:** 10.1530/ETJ-26-0044

**Published:** 2026-07-31

**Authors:** Peter Taylor, Fraser Gibb, Marco Medici, Dhruv Parekh, Carla Moran, Leonidas Duntas, Bijay Vaidya, Colin Dayan, Kristien Boelaert

**Affiliations:** ^1^ Department of Immunology and Immunotherapy, School of Infection, Inflammation and Immunology, College of Medicine and Health, University of Birmingham, Birmingham, UK; ^2^ Department of Endocrinology, University Hospital of Wales, Cardiff, UK; ^3^ Edinburgh Centre for Endocrinology & Diabetes, Royal Infirmary of Edinburgh, Edinburgh, UK; ^4^ Department of Internal Medicine, Erasmus Medical Centre, Rotterdam, Netherlands; ^5^ Birmingham Acute Care Research Group, Institute of Inflammation and Ageing, University of Birmingham, Birmingham, UK; ^6^ School of Medicine, University College Dublin, Dublin, Ireland; ^7^ Evgenideion Hospital, Unit of Endocrinology, Diabetes and Metabolism, Thyroid Section, National and Kapodistrian University of Athens, Athens, Greece; ^8^ Department of Endocrinology, Royal Devon University Hospital|University of Exeter Medical School, Exeter, UK; ^9^ Department of Applied Health, School of Health Sciences, College of Medicine and Health, University of Birmingham, Birmingham, UK

**Keywords:** thyroid emergencies, guidelines, myxoedema coma, hypothyroidism, hypothermia, Society for Endocrinology, British Thyroid Association, European Thyroid Association Welsh Endocrine and Diabetes Society

## Abstract

Myxoedema coma is a rare, life-threatening endocrine emergency resulting from severe hypothyroidism, most commonly affecting older adults. It is characterised by altered mental status, hypothermia, and multiorgan dysfunction, with a high mortality rate if not promptly recognised and treated. This consensus statement provides recommendations for the diagnosis, initial resuscitation, and definitive management of myxoedema coma based on clinician expertise and experience. Key elements include early recognition of precipitating factors, the use of early empiric stress dose glucocorticoids, supportive care in a high-dependency or intensive care setting, and cautious thyroid hormone replacement. Guidance is also given on ventilatory and circulatory support, correction of electrolyte and metabolic disturbances, and monitoring of therapy. The document emphasises a structured approach to diagnosis and management to improve outcomes in this rare but critical condition.

## Introduction

Myxoedema coma is a rare, life-threatening condition characterised by profound hypothyroidism and altered mental status (1, 2). The physician Gull, in 1874, was the first to describe significant hypothyroidism under the term ‘myxoedema’ due to its characteristic features of swollen skin, with the word coma being added later for severe cases with decompensation. Despite its name, most patients are not comatose and the presentation is that of decompensated profound hypothyroidism.

Due to widespread easy access to thyroid function testing, it is very unusual for myxoedema coma to be the first presentation of undiagnosed hypothyroidism. The typical patient is usually an elderly female patient with well-established hypothyroidism (either auto-immune or following definitive treatment for hyperthyroidism), who has poor compliance with levothyroxine with a precipitating event triggering the emergency presentation. Prompt thyroid hormone and glucocorticoid replacement combined with supportive care on a high dependency unit (HDU) or intensive therapy unit (ITU), if appropriate, is key, although mortality may still be as high as 50% (1, 3, 4, 5, 6).

We are aware that many hospitals do not have IV levothyroxine readily available. We recommend that local endocrinology teams liaise with their pharmacology and pharmacy colleagues to ensure availability, given the need for rapid access and its long shelf life.

## Methods

This document represents an expert consensus clinical guidance statement developed following nomination of panel members by the European Thyroid Association, British Thyroid Association, Society for Endocrinology, and Welsh Endocrine and Diabetes Society. Panel members were selected based on recognised expertise in thyroid disease and endocrine emergencies. A structured literature review was undertaken using PubMed/MEDLINE (1990–2025) with search terms including ‘myxoedema coma’, ‘severe hypothyroidism’, ‘thyroid hormone replacement’, and ‘critical care’. Relevant guidelines and key observational studies were reviewed.

Draft recommendations were iteratively revised by the panel until consensus agreement was achieved. Given the rarity of myxoedema coma and limited high-quality trial data, formal Delphi methodology or evidence grading frameworks were not applied. Recommendations therefore reflect integration of available evidence with expert clinical consensus.

## Diagnosis

Key findings that should prompt consideration of myxoedema coma are as follows:

- History of hypothyroidism*.* This may be auto-immune or following previous radioactive iodine treatment or total thyroidectomy for hyperthyroidism.
- Altered mental status*.* Worsening lethargy and somnolence are likely to have been present for weeks and even months. Transient episodes of reduced consciousness may have occurred prior to admission, and less commonly, patients present in coma.
- Hypothermia. This may be profound, and the degree of hypothermia is also predictive of mortality.
- Precipitating event*.* Typically, patients present in the winter months, or following cold exposure, infection, stroke, cardiac event, gastrointestinal bleeding, and certain drugs (such as diuretics, tranquillisers, sedatives, and analgesics).

Examination findings, in addition to hypothermia, include hypoventilation, bradycardia, dry coarse skin, macroglossia, particularly in long-standing primary hypothyroidism, and delayed relaxation of deep tendon reflexes. A potential scoring system (7) is shown in Table 1.

**Table 1:** Possible myxoedema coma scoring system adapted from Popoveniuc *et al.* (7). A score of ≥60 is highly suggestive of myxoedema coma. A score of 45–59 suggests a risk of myxoedema coma. A score lower than 45 makes myxoedema coma unlikely. Whilst a score >60 is highly suggestive of myxoedema coma, similar scores may be observed in other critical illnesses associated with altered mental status and physiological derangement (e.g. sepsis and major cardiovascular events). The scoring system should therefore be interpreted within the clinical context and in conjunction with biochemical evidence of hypothyroidism, rather than used in isolation.

Marker	Points
Thermoregulatory dysfunction (temperature, °C/°F)	
>35/95	0
32–35/89.6–95	10
<32/89.6	20
Central nervous system effects	
Absent	0
Somnolent/lethargy	10
Obtunded	15
Stupor	20
Coma/seizures	30
Gastrointestinal system	
Anorexia/abdominal pain/constipation	5
Decreased intestinal motility	10
Paralytic ileus	15
Precipitating event	
Absent	0
Present	10
Cardiovascular	
Heart rate (bpm)	
≥60	0
50–59	10
40–49	20
<40	30
Others	
Pericardial/pleural effusion	10
Pulmonary oedema	10
Cardiomegaly	15
Hypotension	20
Metabolic	
Hyponatraemia	10
Hypoglycaemia	10
Hypoxaemia	10
Hypercarbia	10
Low GFR	10

GFR, glomerular filtration rate; BPM, beats per minute.

## Laboratory results

Most patients have low/undetectable serum FT4 and very high serum thyroid-stimulating hormone (TSH). However, if there is central hypothyroidism, TSH may be low or normal. Additional laboratory findings include hyponatraemia, hypoglycaemia, hypercholesterolaemia, elevated creatinine kinase, and macrocytic anaemia.

### Treatment

**Figure 1:**
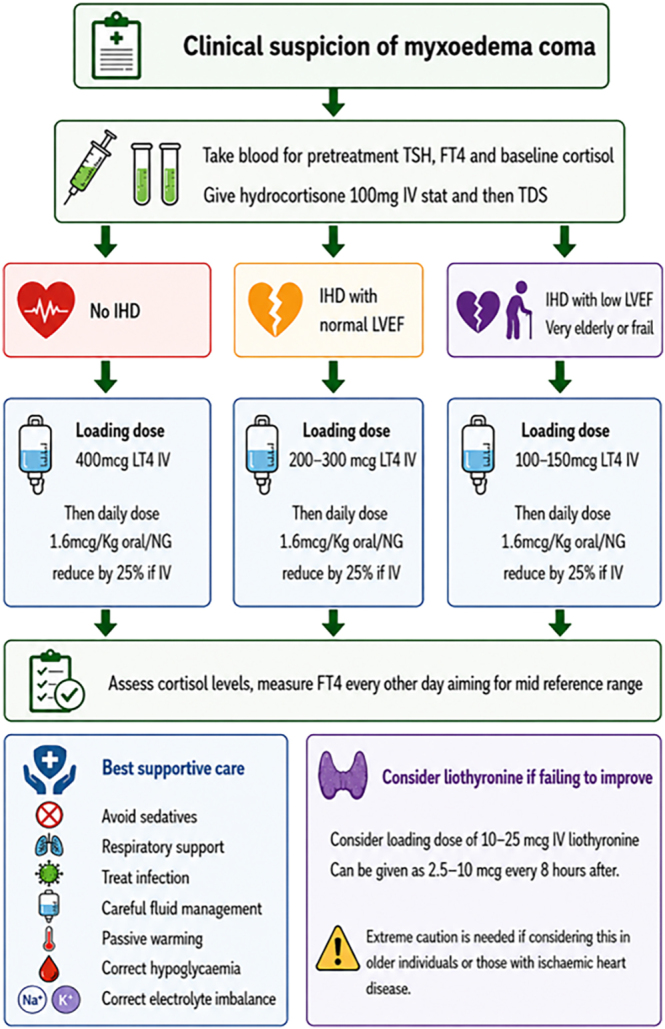
Treatment algorithm of myxoedema coma. FT4, free thyroxine; IV, intravenous; NG, nasogastric; TDS, three times daily; TSH, thyroid-stimulating hormone.

Prompt treatment with thyroid hormone and stress dose glucocorticoid therapy is essential (4). Therapy can and should be instituted based on clinical suspicion alone and should not be unduly delayed whilst waiting for blood test results, although these are usually rapidly available (2). A treatment algorithm is shown in Fig. 1.

Aggressive treatment of the precipitating factor and correction of electrolyte abnormalities are essential. Furthermore, do not assume that impaired conscious level/cardiovascular instability is solely due to the thyroid – the underlying precipitant, e.g. sepsis or electrolyte disturbance, may be the cause.

### Thyroid hormone replacement – key considerations

- The initial dose of levothyroxine requires some consideration: too low a dose may result in failure to reverse the clinical situation, and too high a dose may precipitate a fatal tachycardia or myocardial infarction.
- Treatment with levothyroxine should be IV initially, due to impaired gastrointestinal absorption.
- An initial large IV loading dose of 200–400 μg levothyroxine is recommended (2) with lower doses given for smaller or older patients or those with a history of ischaemic heart disease or significant arrhythmia (2). In these cases, an initial dose of 100–200 μg levothyroxine should be considered.
- Following the loading dose of levothyroxine, a daily replacement dose of 1.6 μg/kg (reduced to 75% whilst been given IV) is recommended (2).
- The role of liothyronine (LT3) is controversial. Owing to severe illness, conversion of FT4 to FT3 may be impaired and this may prevent reversal of myxoedema coma. However, too high a dose of liothyronine may trigger fatal cardiac complications (7). If LT3 is being used, consider 2.5–10 μg every 8 h. A pragmatic approach may be to just utilise levothyroxine in the first 24 h and to utilise liothyronine only in patients who are failing to improve. Extreme caution is needed if considering this in older individuals or those with established ischaemic heart disease.
- An alternative strategy used by some clinicians is an initial IV dose of 200–300 μg levothyroxine plus 10–25 μg IV liothyronine, followed by 2.5–10 μg IV liothyronine every 8 h with lower doses in older patients and in the presence of cardiovascular risk factors (1).

### Glucocorticoid treatment

- Restoration of normal metabolic rate may trigger an Addisonian crisis in individuals with co-existing adrenal insufficiency. Hydrocortisone IV 100 mg stat and then 50 mg QDS (or 200 mg over 24 h via a syringe driver in HDU/ITU environments) are recommended in all patients. However, the majority of patients will not have adrenal insufficiency; therefore, steroids can be discontinued as soon as the adrenal axis has been evaluated and cleared. This can be done when initial blood results (pre-treatment) are available or by 09:00 AM serum cortisol or cosyntropin (Synacthen) test undertaken prior to morning steroid dose.

### Warming

- Passive warming with blankets is recommended. Patients are often maximally vasoconstricted, so active peripheral warming may trigger cardiovascular collapse and should be avoided.

### Precipitating factor treatment

- Try to identify the precipitating factor. It is worth highlighting that typical signs of infection, such as fever and tachycardic and elevated white cell count, may be absent. Prophylactic broad-spectrum antibiotics are, therefore, recommended until infection can be satisfactorily ruled out.

### Hypoventilation

- These individuals are at an increased risk of requiring mechanical ventilation, and patients should be monitored closely for this.

### Hypotension

- Cautious volume expansion with IV crystalloid/blood should be utilised for hypotension. If hyponatraemia is present, this will also need monitoring closely.

### Hypoglycaemia

- Hypoglycaemia is common and needs to be monitored for closely, and patients may need dextrose infusions.

### Ongoing management

Once a patient’s cardiovascular and respiratory status has stabilised, liothyronine, if it has been utilised, can be stopped and replaced by additional levothyroxine. Intravenous thyroid hormones can be replaced by oral administration once the patient is fully conscious. Alternate-day thyroid function tests aiming for FT4 levels to be in the middle of the reference range without excess FT3 levels are key. TSH will take some time to normalise, so it cannot be used to guide treatment response. Treatment may need to be aggressive if the patient is failing to improve with increasing doses of levothyroxine and/or initiation of liothyronine.

Case reports have identified that patients may develop acute kidney injury, gastrointestinal bleeding, and even pulmonary haemorrhage after initial stabilisation, so ongoing careful monitoring is required for these (3, 6).

## Declaration of interest

The document should be considered as an expert consensus only; it is not intended to determine an absolute standard of medical care. The clinical team concerned must make the management plan fully considering the individual circumstances of each patient. The authors declare that there is no conflict of interest that could be perceived as prejudicing the impartiality of the work reported.

## Funding

This work did not receive any specific grant from any funding agency in the public, commercial, or not-for-profit sector.
